# Assessing Bone Mineral Density in Weight-Bearing Regions of the Body through Texture Analysis of Abdomen and Pelvis CT Hounsfield Unit

**DOI:** 10.3390/diagnostics13182968

**Published:** 2023-09-16

**Authors:** Min Woo Kim, Jung Wook Huh, Young Min Noh, Han Eol Seo, Dong Ha Lee

**Affiliations:** Department of Orthopedic Surgery, Busan Medical Center, 62, Yangjeong-ro, Busanjin-gu, Busan 47227, Republic of Korea; drkimminwoo@naver.com (M.W.K.); gizer00@hanmail.net (J.W.H.); doctornoh77@naver.com (Y.M.N.); mdseo86@gmail.com (H.E.S.)

**Keywords:** texture analysis, bone mineral density (BMD), osteoporosis screening, computed tomography, Hounsfield units

## Abstract

**Objective**: This study aimed to develop a novel method for opportunistically screening osteoporosis by measuring bone mineral density (BMD) from CT images. We addressed the limitations of commercially available software and introduced texture analysis using Hounsfield units (HU) as an alternative approach. **Methods**: A total of 458 samples (296 patients) were selected from a dataset of 1320 cases (782 patients) between 1 March 2013, and 30 August 2022. BMD measurements were obtained from the ilium, femoral neck, intertrochanteric region of both femurs, and L1–L5 and sacrum spine body. The region of interest (ROI) for each patient’s CT scan was defined as the maximum trabecular area of the spine body, ilium, femoral neck, and femur intertrochanter. Using gray-level co-occurrence matrices, we extracted 45 texture features from each ROI. Linear regression analysis was employed to predict BMD, and the top five influential texture features were identified. **Results**: The linear regression (LR) model yielded correlation coefficients (R-squared values) for total lumbar BMD, total lumbar BMC, total femur BMD, total femur BMC, femur neck BMD, femur neck BMC, femur intertrochanter BMD, and femur intertrochanter BMC as follows: 0.643, 0.667, 0.63, 0.635, 0.631, 0.636, 0.68, and 0.68, respectively. Among the 45 texture features considered, the top five influential factors for BMD prediction were Entropy, autocorrelate_32, autocorrelate_32_volume, autocorrelate_64, and autocorrelate_64_volume.

## 1. Introduction

Osteoporosis is a prevalent and consequential condition, causing degradation of bone microarchitecture, notably in the hip and spine; weakened bone durability; and increased susceptibility to osteoporotic fractures [1]. With an increase in life expectancy, the incidence of osteoporosis is projected to continue to rise [2]. Despite the high morbidity rate among patients with osteoporosis, diagnosis and treatment options remain inadequate [3,4,5].

Central dual-energy X-ray absorptiometry (DXA) is currently the preferred method for measuring bone mineral density (BMD) in the lumbar vertebrae and femoral bone, and is widely considered as the reference standard for diagnosing osteoporosis [6,7,8]. However, the accuracy of BMD scans using lumbar DXA can be affected by certain factors such as lumbar degenerative diseases, which can result in higher BMD values [9,10,11,12]. Although quantitative computed tomography (QCT) has the potential to provide a more precise measurement of BMD, the standard for diagnosing osteoporosis based on DXA T-score, as established by the World Health Organization (WHO), remains widely accepted within the medical community.

DXA screening for osteoporosis has been deemed insufficient due to the condition’s asymptomatic nature until substantial incidental fragile fractures, like spine or femur neck fractures, take place [10]. Patients frequently lack an understanding of the gravity of the condition, resulting in low levels of voluntary participation in screening programs [13]. Consequently, there is an increasing agreement regarding the necessity for alternative screening methods to tackle the limitations and underuse of DXA as a screening tool for osteoporosis.

Abdomen–pelvic computed tomography (APCT) is one such examination that is frequently conducted on adults during routine health check-ups or during follow-ups to a disease diagnosis, to evaluate various conditions. If a small proportion of these scans could be leveraged for osteoporosis screening, the impact could be significant. Several studies have demonstrated promising outcomes in the opportunistic screening of osteoporosis using APCT [11,12].

In the recent literature, there has been increasing interest in the potential of computed tomography (CT) as a diagnostic tool for osteoporosis, with some studies covering the entire or partial spine [14,15,16]. Furthermore, there has been a growing interest in using CT attenuation values in the trabecular bone, Hounsfield units (HU) as a complementary method for evaluating BMD using CT. CT measurements offer several benefits over DXA BMD, such as the ability to avoid degenerated areas and select cancellous bones that are more sensitive to osteoporosis. A research study has shown that CT HU values can be used as a reliable diagnostic tool for osteoporosis without relying on DXA BMD [17].

Opportunistic CT screening for osteoporosis has the potential to serve as a valuable diagnostic tool by increasing screening rates without adding to radiation exposure or patient costs. Moreover, the use of stored databases of CT scans conducted for other clinical purposes can allow for automated screening of undiagnosed patients, thus streamlining their management. However, commercializing CT screening has been challenging due to the lack of sufficient evidence supporting its accuracy in identifying the disease. The lack of sufficient evidence supporting CT screening accuracy is partly due to technical limitations in earlier studies, which did not consider other important statistical features to identify osteoporosis using CT scans [13,14,15,16].

The evolution of osteoporosis diagnosis has been significantly marked by the rise of quantitative imaging techniques based on radiomics. One of the prevailing challenges in this realm is the ongoing need to develop techniques that minimize manual interventions, specifically the segmentation of bone regions. It is equally essential to reduce the observer dependency inherent in these diagnostic methods. To bridge these challenges, our study delves deep into the potential of newer techniques, particularly those leveraging deep learning algorithms [18]. These algorithms, by design, bypass the requirement for manual segmentation, and automatically extract critical features from CT scans [19]. Such advancements, we believe, not only make the diagnosis less observer-dependent but can also enhance accuracy and consistency.

Our primary motivation was to investigate the feasibility of using CT texture analysis as a quantitative tool for assessing bone conditions. This led us to compare the effectiveness of CT texture analysis against DXA, which is currently lauded as the gold standard for osteoporosis screening. In our quest for precision, we employed model-based feature extraction techniques on HU values derived from CT scans. We then embarked on analyzing the accuracy of Bone Mineral Density (BMD) predictions using a combination of these features. To further this, linear regression became our tool of choice to project BMD, with our findings pinpointing the top 5 texture features (out of a total of 45 examined) as significant influencers in BMD prediction.

## 2. Materials and Methods

### 2.1. Patient’s Enrollment

The selection and meticulous curation of the dataset are pivotal and ensure our study’s relevance and validity.

Scale and Diversity: Originating from the Busan Medical Center, Busan, Korea, our dataset spans from 1 March 2013 to 30 August 2022. Within this timeframe, we evaluated 1156 cases involving 590 patients, all of whom underwent both CT and DXA scans. Such extensive and diverse data ensure that our conclusions can be generalized beyond a narrow subgroup, rendering our research findings more universally applicable.

Strict Quality Assurance: Our commitment to data quality is evidenced by our rigorous selection criteria. By focusing on patients with a CT and DXA scan interval of less than a month, we effectively minimized potential external influences on bone density. This detailed approach assures our audience of the authenticity and accuracy of our results. Comprehensive Examination Regions: The meticulous inclusion criteria, emphasizing the lumbar, sacrum body axial cuts, and specific femur regions, show our study’s dedication to precision. This focus promises a consistent dataset, pivotal for reliable interpretation and analysis.

Meticulous Exclusion Criteria: The integrity of our dataset is further enhanced by our stringent exclusion parameters. By removing cases with potential confounding factors such as prior fractures or surgeries, we ensure that our conclusions are drawn from a refined and pertinent data pool. Upholding Ethical Standards: Our adherence to the ethical guidelines is reinforced by the approval from the Busan Medical Center Institutional Review Board (IRB 2023-01-001). This adherence not only ensures the rights and safety of our study participants but also adds a layer of moral credibility to our research findings.

**Figure 1:**
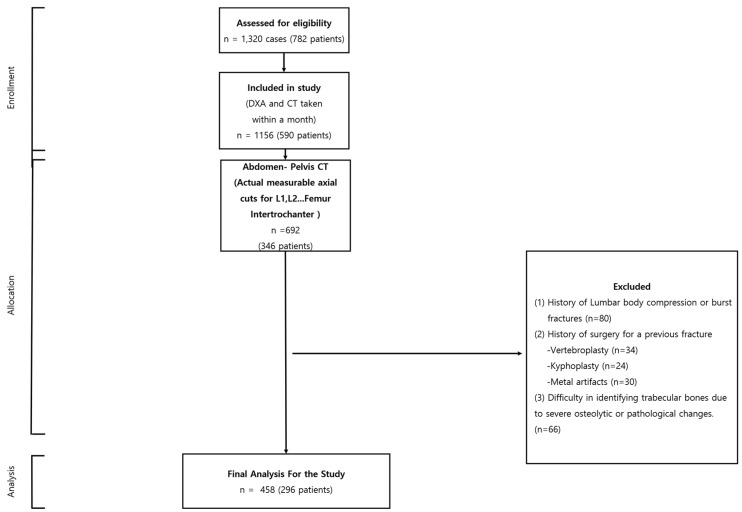
Flowchart showing the selection of cases.

In summation, our dataset, characterized by its vastness, precision, and ethical underpinnings, serves as the backbone of our research, allowing us to make significant contributions to the discourse on bone mineral density assessment. After applying the exclusion criteria, the analysis was performed on 458 cases, which belonged to 296 patients (Figure 1).

### 2.2. Imaging Protocols for CT

CT imaging was performed using a Siemens (SOMATOM 128, Definition AS+) scanner (Siemens Healthcare, Forchheim, Germany) with a single-energy CT protocol. The parameters used included 120 kVp, 247 mA, dose modulation, 0.6 mm collimation, and an effective pitch of 0.8. The resulting images had a slice thickness of 3.0 mm for both the abdomen and pelvis CT, and a 3.0 mm slice increment.

### 2.3. Imaging Protocols for DXA

Routine clinical examination at our institution involved obtaining DXA measurements using a standard DXA device (GE Lunar Prodigy, GE Healthcare, Chicago, IL, USA) and following a protocol for DXA imaging. The generated DXA images were analyzed automatically, and reports were produced using software ver. 1.1 provided by the vendor (Physicians Report Writer DX, Hologic, Discovery Wi, Chicago, IL, USA).

### 2.4. Bone Images

**Figure 2:**
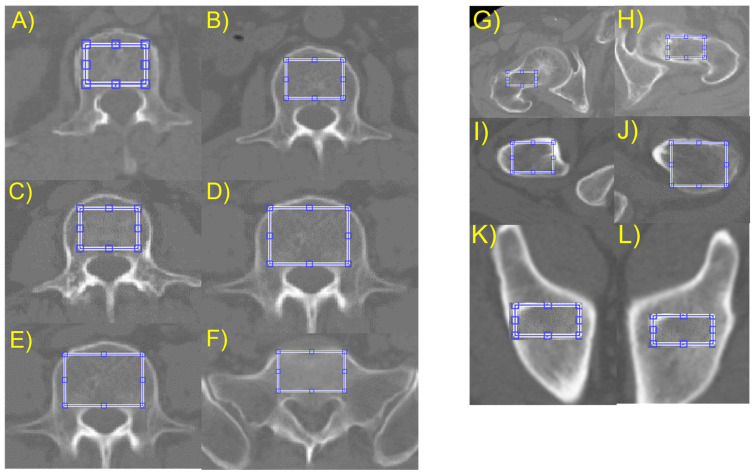
Regions of Interest in axial cuts of abdomen (**left**) and pelvis (**right**) CT. (**A**) Lumbar vertebra 1, (**B**) lumbar vertebra 2, (**C**) lumbar vertebra 3, (**D**) lumbar vertebra 4, (**E**) lumbar vertebra 5, (**F**) sacrum vertebra 1, (**G**) femur neck right, (**H**) femur neck left, (**I**) femur intertrochanter right, (**J**) femur intertrochanter left, (**K**) acetabulum right, and (**L**) acetabulum left.

To measure the bone images statistically, the researchers selected regions of interest (ROIs) that were restricted to the cancellous bone to avoid measurement distortions caused by other parts. To isolate these ROIs, the thresholding method was chosen from several available methods. A 2D slice image that contained the maximum axial cancellous bone area of the lumbar body, ilium, and proximal femur was selected from the CT cuts of each patient. A rectangular region covering most of the trabecular area was chosen for texture analysis, as shown in Figure 2.

### 2.5. Feature Acquisition

**Figure 3:**
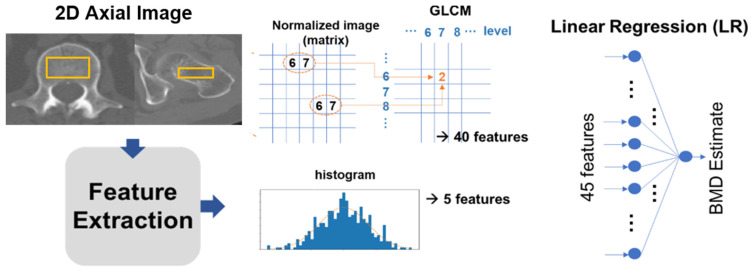
Schematic flow outlining the steps involved in estimating bone mineral density (BMD) using computed tomography (CT) imaging.

The study extracted 45 features from the ROIs. Five of these were intensity-based, sourced from a histogram, while the remaining 40 were texture-based features retrieved using a gray-level co-occurrence matrix (GLCM). These features were then channeled as input for a linear regression (LR) model, which estimated the BMD through a linear combination of the 45 input values. From the image histogram of the ROI, we procured intensity-based features which included parameters like the mean, standard deviation, skewness, kurtosis, and entropy. These features exemplify bone intensities such as brightness, asymmetry, randomness, uniformity, and sharpness. Moreover, from the GLCM, 40 texture-based features were harnessed to offer insights about the spatial relations between adjacent pixels in a 2D image (as depicted in Figure 3).

The features were extracted from the GLCM with a matrix size of *n* × *n*. Here, the (i,j) element equated to the total times two horizontally adjacent pixels which had values i and j in the discretized and normalized ROI image, with an intensity scale that varied from 1 to *n*. For every sample ROI image, eight GLCMs were created using four levels (*n* = 16, 32, 64, 128) and two orientations (horizontal and vertical), and five statistics (entropy, contrast, correlation, homogeneity, and variance) were measured for each GLCM. To ensure model robustness, we partitioned our dataset into training and test subsets. The training subset aided in the model’s training, while the test subset evaluated its efficacy. As elucidated in Figure 3, the model employs a single hidden-layer fully connected network with one output.

### 2.6. Correlation Analysis of BMD

Statistical analyses were performed using MATLAB 9.10 R2021a (MathWorks, Natick, MA, USA). The study utilized a multiple regression model to correlate between estimated BMD values and reference values obtained through dual-energy X-ray absorptiometry (DXA). The features used in the models were denoted as xij, where i represents the feature number and j represents the sample (case) number. The values of xij were normalized using the sample mean and standard deviation, denoted as xi and σi, respectively. Similarly, the reference BMD values, denoted as yj, were normalized using the sample mean and standard deviation, denoted as y and σ, respectively.

To predict BMD estimates, the LR model relied on a weighted combination of 45 features along with a single bias term, represented as yj = w0 + twixij. The weights, wi, were obtained by optimizing the model to reduce the difference between the predicted BMD estimates and the actual BMD values in the dataset, e = jyj − yj2. The study did not employ regularization techniques and did not separate the dataset into training and test subsets.

### 2.7. BMD Correlation Max Index 5 Features

**Figure 4:**
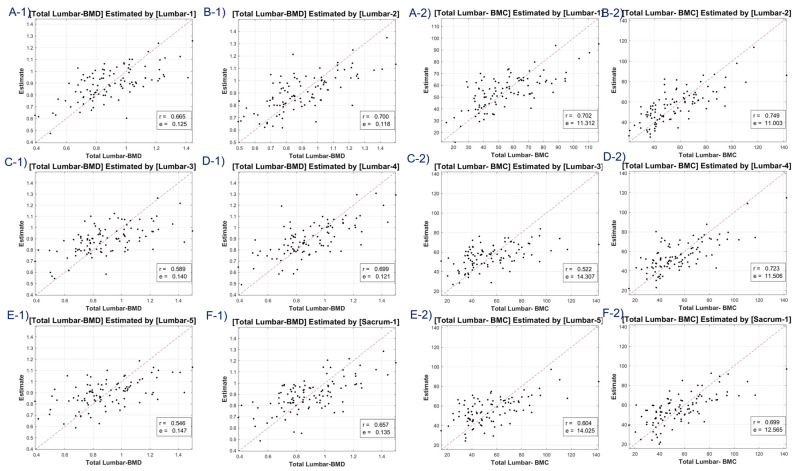
Correlation coefficient and mean squared error of total lumbar BMD—estimates (**A-1**–**F-1**) and total lumbar BMC—estimates (**A-2**–**F-2**) using linear regression model. Letters A–F are regions defined in Figure 2.

In our analysis of the dataset, we identified five maximum index features that exhibited the strongest correlation with BMD values. These features were selected through correlation analysis, and we used Pearson’s correlation coefficient to measure the relationship between each feature and BMD. Specifically, we applied the coding corr(feature(idx), BMD) for idx = 1, 2, …, 45, adjusting it for the different femurs in the dataset (femur 1, femur 2, etc.). The five features identified were 5, 10, 15, 20, and 25, with corresponding Pearson correlation coefficients ranging from entropy, autoco-32, autoco-64, autoco128, and energy (Figure 4). These results highlight the potential importance of these specific features in understanding the relationship between bone texture and BMD and may have important implications for the diagnosis and treatment of bone diseases.

## 3. Results

### 3.1. Patient Demographics

This study involved 296 patients, of whom 124 were male and 172 were female. The average age of the patients was 58.40 ± 11.68 years. We analyzed the data obtained from the patients’ CT and DXA scans, with a mean gap of 2.57 ± 3.54 days between the two procedures. Additionally, we recorded the patients’ BMI, which had a mean value of 23.76 ± 4.75 kg/m^2^.

### 3.2. Correlation Test

**Figure 5:**
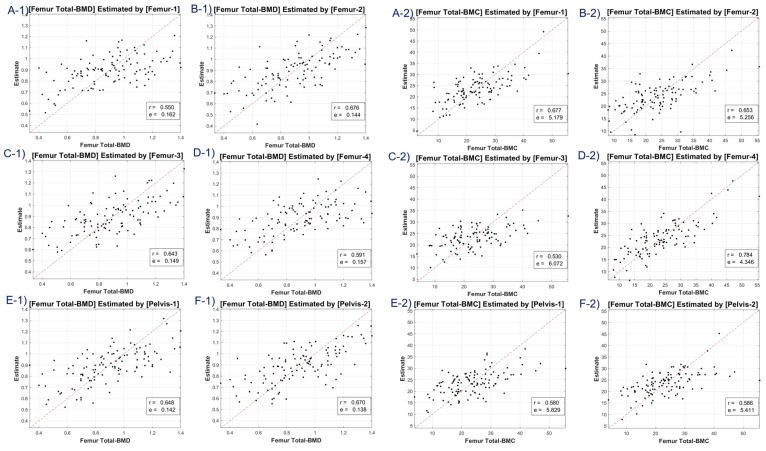
Correlation coefficient and mean squared error of total femur BMD—estimates (**A-1**–**F-1**), and total femur BMC—estimates (**A-2**–**F-2**) using linear regression model. Letters A–F are regions defined in Figure 2.

**Figure 6:**
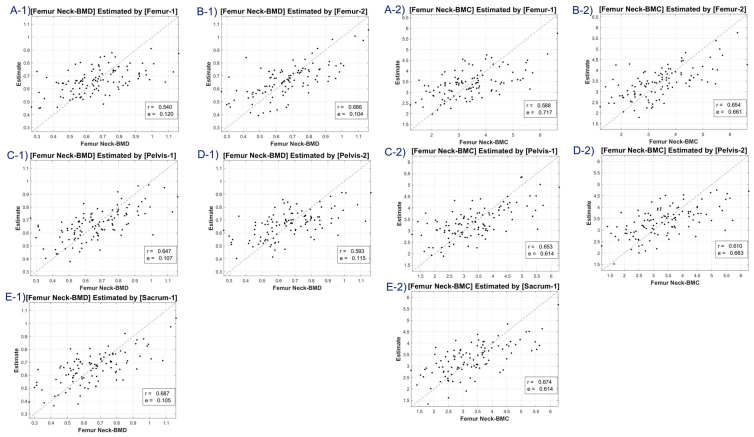
Correlation coefficient and mean squared error of femur neck BMD—estimates (**A-1**–**E-1**), and femur neck BMC—estimates (**A-2**–**E-2**) using linear regression model. Letters A–E are regions defined in Figure 2.

**Figure 7:**
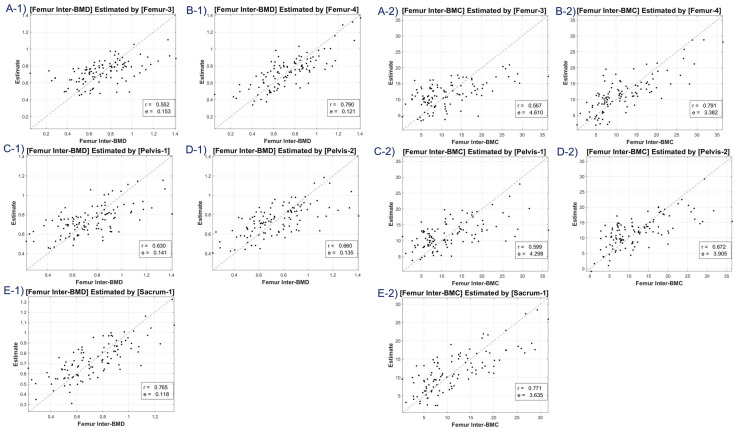
Correlation coefficient and mean squared error of femur intertrochanter BMD—estimates (**A-1**–**E-1**), and femur intertrochanter BMC—estimates (**A-2**–**E-2**) using linear regression model. Letters A–E are regions defined in Figure 2.

Our study included scatter plots (Figure 4, Figure 5, Figure 6 and Figure 7) showing the predicted BMD values versus the actual BMD values, across all cases. These plots depict the estimated values for the test samples, which were predicted by the neural network after it was trained on the training samples. Table 1 presents the correlation coefficient and mean squared error (MSE) for the predicted and reference bone mineral density (BMD) values. The LR model was used to analyze datasets of total lumbar BMD, total lumbar BMC, total femur BMD, total femur BMC, femur neck BMD, femur neck BMC, femur intertrochanter BMD, and femur intertrochanter BMC. The resulting correlation coefficients and MSE values were 0.643 (0.131), 0.667 (12.453), 0.63 (0.149), 0.635 (5.349), 0.631 (0.11), 0.636 (0.654), 0.68 (0.134), and 0.68 (3.97), respectively. The performance of a conventional method (mean HU feature) was compared with our method (45 features) in Table 1. Our LR model using all 45 features provided significantly better scores for estimating the average attenuation coefficient.

### 3.3. Max Index (5) Counting

**Figure 8:**
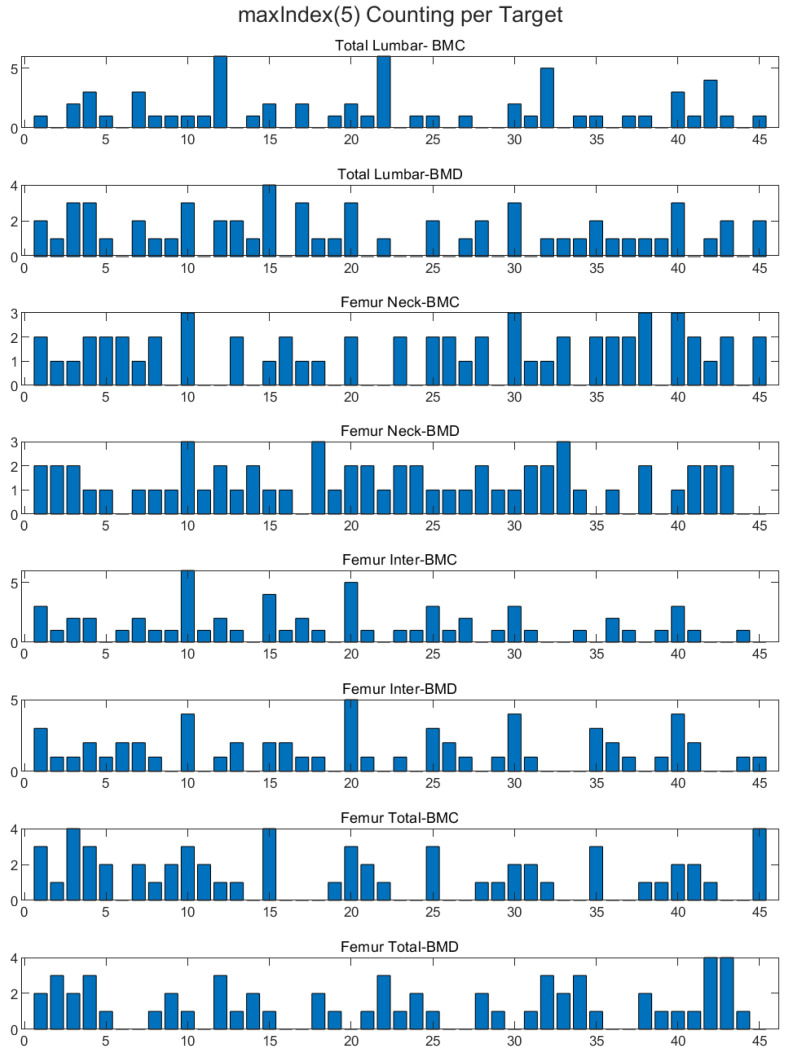
Max index (5) counting per target. The *X*-axis represents the 45 features, and the *Y*-axis depicts the correlation coefficient between the number of features and the weight-bearing regions of BMD or BMC.

The max index (5) per target for 45 features was counted to find the most powerful influence on the BMD estimation and correlation. It was determined that 10, 15, 20, 25, and 35 were the highest max index 5 features for targeting, referring to entropy, autocorrelate_32, autocorrelate_32_ volume, autocorrelate_64, autocorrelate_64_volume, and autocorrelate_128_volume (Figure 8).

## 4. Discussion

The study utilized CT HU texture analysis to develop an LR model that incorporated 45 features for BMD estimation. The correlation between the predicted BMD values and the actual BMD values was evaluated. The LR model was applied to eight BMD/BMC targets, including total lumbar BMD, total lumbar BMC, total femur BMD, total femur BMC, femur neck BMD, femur neck BMC, femur intertrochanter BMD, and femur intertrochanter BMC. The correlation coefficients ranged from 0.63 to 0.68, indicating moderate to strong positive correlations between the estimated and reference values. The MSE values ranged from 0.11 to 12.453, with the lowest value observed for femur neck BMD and the highest value observed for total lumbar BMC. Table 1 compares the performance of the LR model using the 45 features to a conventional method that uses a mean Hounsfield unit (HU) feature. The LR model using all 45 features provided better scores for the average attenuation coefficient, indicating better performance compared to the conventional method.

This study’s main strength lies in its robustness, which is demonstrated by the use of a large sample size including both male and female patients, as well as a shorter scan interval (2.57 ± 3.54) between CT (abdomen and pelvis) and DXA compared to previous studies [19]. The texture analysis of CT HU values yielded models that estimated BMD values with a strong correlation to the DXA-based BMD. In general, there are several approaches that can be used to identify the most important features in a LR model, including: The coefficient magnitude: The magnitude of the coefficients in the LR model can be used to determine which features have the strongest influence on the outcome. Features with larger coefficient values are considered more important [20]. Recursive Feature Elimination (RFE): This is an iterative process that involves training the LR model on subsets of the features and selecting the subset that provides the best performance. The features that are included in the best-performing subset are considered the most important [21]. Correlation-based feature selection: This approach involves selecting features that have the highest correlation with the outcome variable [22]. Features with high correlation are considered more important. Lasso regularization: The method used in this study employs a penalty term to promote sparsity in the LR model, favoring solutions that use only a subset of the available features [23]. The Lasso method can be used to identify the subset of features that have the strongest influence on the outcome. We adopted a correlation-based feature selection for max 5 index targeting.

Based on the results, entropy is a measure of the randomness or disorder in an image, and can be used to quantify the texture or complexity of bone tissue [24]. Autocorrelation is a statistical technique that measures the similarity between adjacent pixels or voxels in an image, and can be used to characterize the spatial structure of bone tissue [25]. Other features that are commonly used in medical image analysis include intensity-based features (e.g., mean, median, standard deviation), shape-based features (e.g., volume, surface area), and histogram-based features (e.g., skewness, kurtosis). The specific features that are most informative for a given application will depend on the imaging modality, the type of tissue or pathology being studied, and the specific research question being addressed.

Texture analysis is a powerful tool for quantifying the microstructural properties of bone images. It enables the extraction of information on the pattern and arrangement of variations in pixel intensities over a small region of interest. Texture analysis involves the application of various mathematical algorithms to extract information on the patterns and distribution of pixel values within an image. These algorithms include fractal analysis, run-length distribution analysis, and Markov random field-based models, among others. Fractal analysis has been used to identify patients with osteoporosis by analyzing the texture of X-ray images. Run-length distribution analysis has been used to assess bone loss in rat models. Markov random field-based models, such as semi-variograms, have been used to evaluate bone characteristics in recent studies. Overall, texture analysis is an important tool for analyzing bone images and can provide valuable information for the diagnosis and treatment of bone-related conditions [26,27].

Quantitative CT (QCT) is a promising alternative to DXA, as it can overcome many of its limitations [28]. While QCT is used in clinical settings, it is not yet widely adopted due to its limited applicability, which is restricted to the spine, as well as its requirement for phantom calibration, its high cost, and its high radiation hazard. On the other hand, conventional CT scans are widely available and commonly used in medical imaging, indicating the potential for detecting osteoporosis using this modality. Given the fact that large image databases are already available in clinical institutions, it is possible to identify disorders in CT images that were acquired for other clinical purposes. The objective of our study was to create an initial model for estimating BMD in peripheral bones like the clavicle and ulna, which are not typically included in DXA scans. Nonetheless, we observed a lower correlation value for the femoral neck than for the lumbar or femoral intertrochanter regions, indicating that further investigation is needed to understand the observed discrepancies between the selected bone regions [2,10,11,29,30].

Our research integrated an LR model, leveraging the potent capabilities of deep learning, which has revolutionized prediction generation across various sectors. Medical imaging, in particular, has reaped immense benefits from machine learning due to its potential to augment diagnostic precision. The discipline of radiomics, which delves into the extraction of quantitative features from image regions of interest (ROIs), is becoming an increasingly sought-after method to fulfill prognostic or predictive objectives. We meticulously adhered to the traditional radiomics pathway, which encompassed pre-processing, manual segmentation, and feature extraction. It is noteworthy that our feature extraction was grounded in intensity analysis, using metrics such as energy, kurtosis, and skewness. Furthermore, we employed texture analysis hinged on the gray-level co-occurrence matrix (GLCM), a widely acclaimed model instrumental in extracting features connected to tissue heterogeneity [31].

A discernible limitation emerged in the LR model’s tendency to introduce substantial bias in boundary BMD ranges, as depicted in Figure 4, Figure 5, Figure 6 and Figure 7. Addressing this concern, we delved into an in-depth analysis and ascertained that LR models, by design, incline towards minimizing the mean squared error (MSE). This predisposition can potentially skew results, especially when classifying latent osteoporosis cases where a judicious equilibrium between sensitivity and specificity is imperative. Our systematic criteria setting for the deep-learning cutoff, which precluded cases bereft of even one feature value or where DXA BMD was below 0.15, culminated in generating an exemplary array of correlation values. These consistently hovered around or exceeded 0.63, bolstering our belief in CT HU texture analysis as a reliable predictor of BMD in the foreseeable future.

Critically, while our study focused on the Siemens SOMATOM 128, we designed our methodology to be vendor neutral. This means the extracted features and the subsequent analysis should be coherent across CT images, independent of the machine manufacturer. Nonetheless, recognizing the potential nuances introduced by diverse CT protocols and hardware, our future trajectory is geared towards endorsing the efficacy of our technique across a spectrum of CT devices, ensuring its robustness and universal relevance.

It is important to note that our study has some limitations that should be considered when interpreting the results. We did not compare the predicted values of CT-derived BMD with those of QCT, the recognized gold standard for BMD measurement. Despite the broader availability of CT, validating our findings requires further studies that directly compare the two methodologies. A 3D texture analysis spanning the entire volume of interest might offer a more nuanced and accurate assessment. Nonetheless, this approach is notably more intricate and labor-intensive than our study’s 2D analysis.

Our participant pool primarily consisted of an Asian cohort from a singular center, potentially limiting the applicability of our findings across diverse global populations. We acknowledge the differing manifestations of osteoporosis between genders. The current study did not particularly segment the data based on these gender-specific characteristics, which might influence the comprehensive applicability of our conclusions. This dimension offers a fertile ground for future research endeavors. Relying on handcrafted features might have circumscribed further enhancements in predictive accuracy. Prospective studies might consider deploying deep-learning techniques like convolutional neural networks, offering richer feature extraction from the ROI images.

## 5. Conclusions

Our study demonstrates the efficacy of morphometric texture analysis using CT Hounsfield units (HU) as an additional diagnostic tool for osteoporosis screening. While addressing the limitations of commercially available software, our method provides Supplemental Information to DXA measurements for improved diagnosis and assessment.

## Figures and Tables

**Table 1:** Correlation coefficient and mean squared error of the LR model.

Model	Datasets	Correlation Coefficient (CC)	Mean Squared Error (MSE)
		45 Values	45 Values
**Linear Regression**	Total Lumbar BMD	0.643	0.131
	Total Lumbar BMC	0.667	12.453
	Total Femur BMD	0.63	0.149
	Total Femur BMC	0.635	5.349
	Femur Neck BMD	0.631	0.11
	Femur Neck BMC	0.636	0.654
	Femur Intertrochanter BMD	0.68	0.134
	Femur Intertrochanter BMC	0.68	3.97

## Data Availability

The datasets generated and/or analyzed during the current study are not publicly available because we did not obtain authorization from the patients for disclosure regarding patient privacy. However, datasets are available from the corresponding author on reasonable request.

## Supplementary Materials

The following supporting information can be downloaded at: https://www.mdpi.com/article/10.3390/diagnostics13182968/s1.
