# Supplementary material for: Assessing Bone Mineral Density in Weight-Bearing Regions of the Body through Texture Analysis of Abdomen and Pelvis CT Hounsfield Unit
Source: Diagnostics (Basel). 2023 Sep 16;13(18):2968. doi: 10.3390/diagnostics13182968 (PMC10529497; doi:10.3390/diagnostics13182968)
Supplement: Supplementary file 1 [file diagnostics-13-02968-s001.zip › Figure S1.pdf]

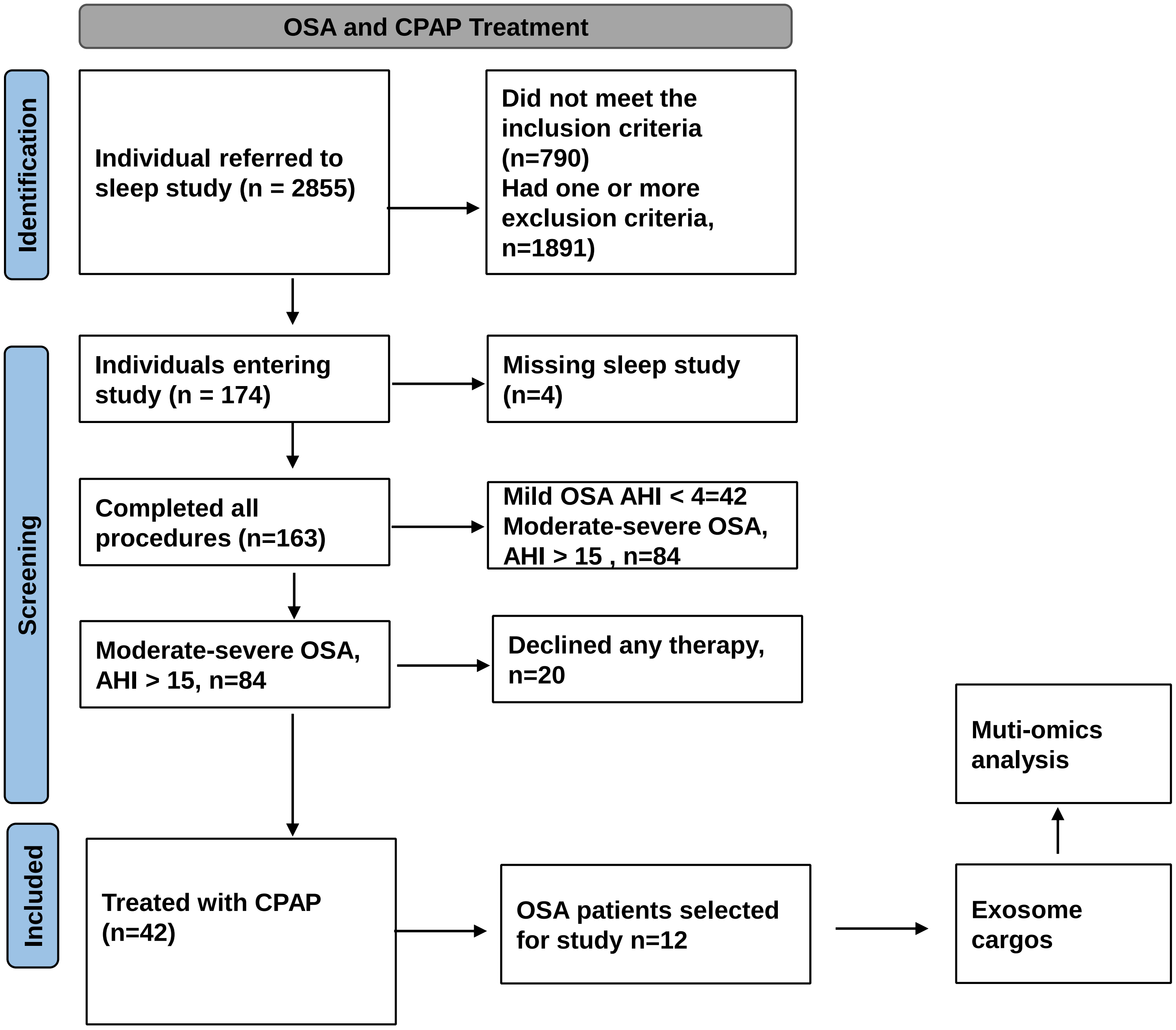
OSA and CPAP Treatment
Identification
Individual referred to sleep study (n = 2855)
Did not meet the inclusion criteria (n=790)
Had one or more exclusion criteria, n=1891)
Screening
Individuals entering study (n = 174)
Missing sleep study (n=4)
Completed all procedures (n=163)
Mild OSA AHI < 4=42
Moderate-severe OSA, AHI > 15 , n=84
Moderate-severe OSA, AHI > 15, n=84
Declined any therapy, n=20
Included
Treated with CPAP (n=42)
OSA patients selected for study n=12
Exosome cargos
Muti-omics analysis
