# Supplementary figures and images for: Assessing Bone Mineral Density in Weight-Bearing Regions of the Body through Texture Analysis of Abdomen and Pelvis CT Hounsfield Unit
Source: Diagnostics (Basel). 2023 Sep 16;13(18):2968. doi: 10.3390/diagnostics13182968 (PMC10529497; doi:10.3390/diagnostics13182968)

## Clinical on Comp 1

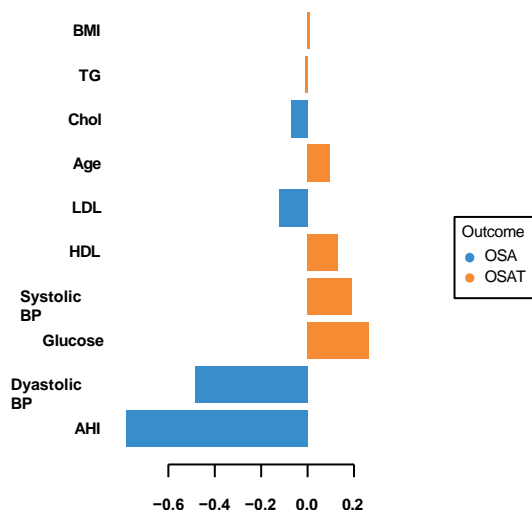

## Lipids on Comp 1

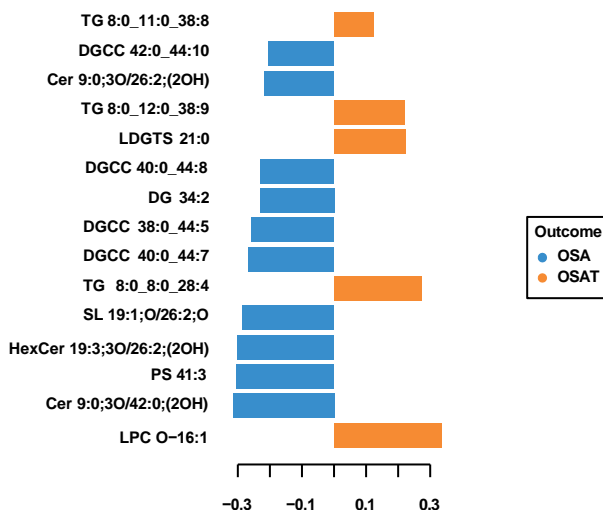

## Proteomics on Comp 1

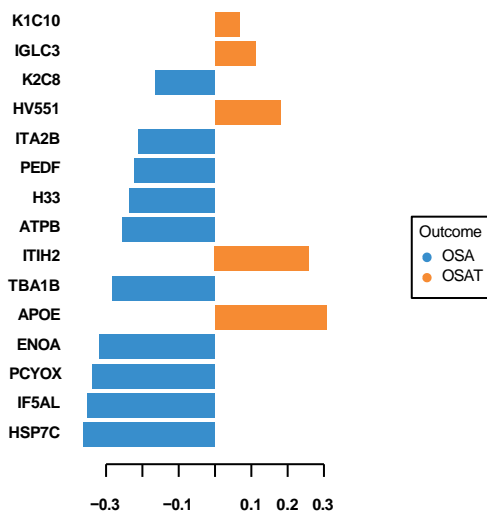

## miRNAs on Comp 1

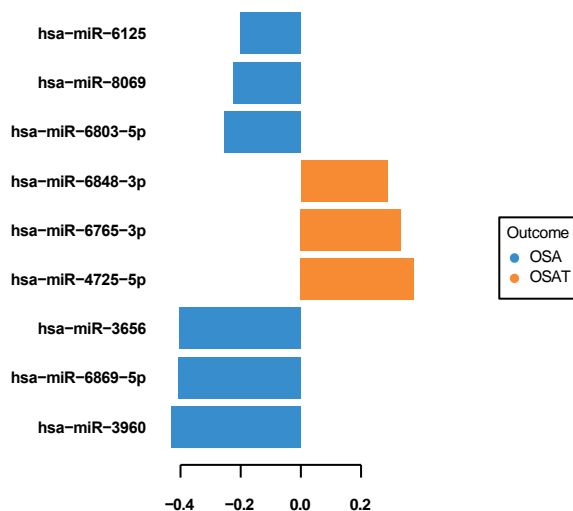

Supplement: Supplementary file 1 [file diagnostics-13-02968-s001.zip › Figure S10.pdf]

## Clinical

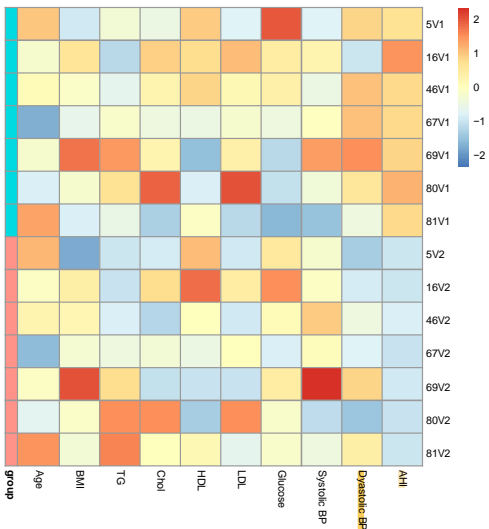

## Lipids

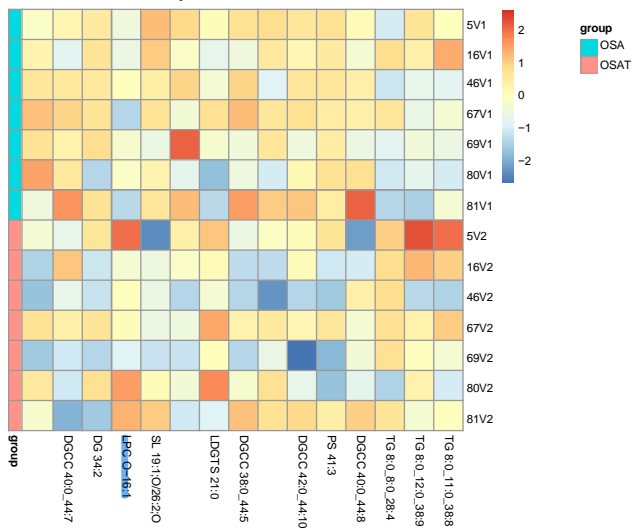

## Proteomics

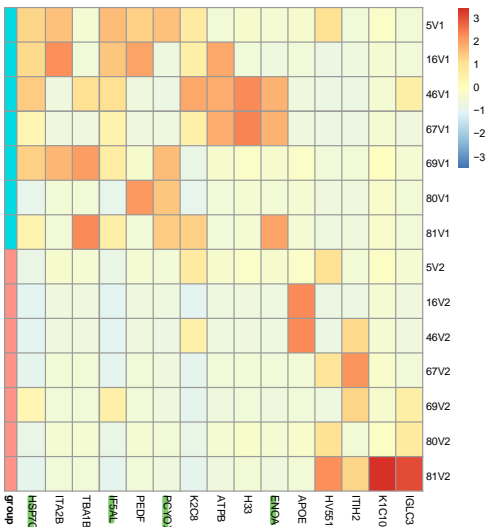

## MiRNAs

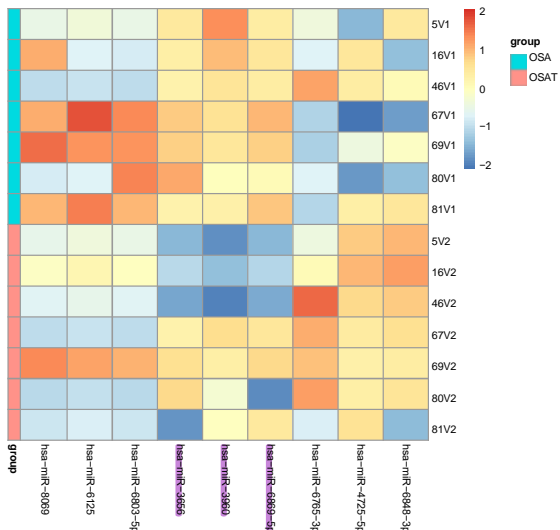

Supplement: Supplementary file 1 [file diagnostics-13-02968-s001.zip › Figure S11.pdf]

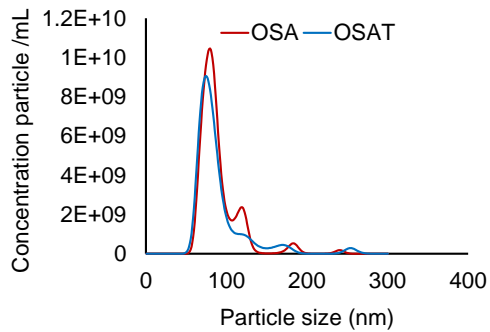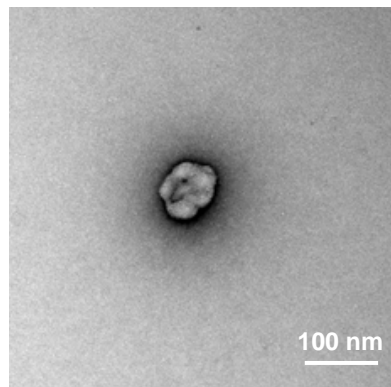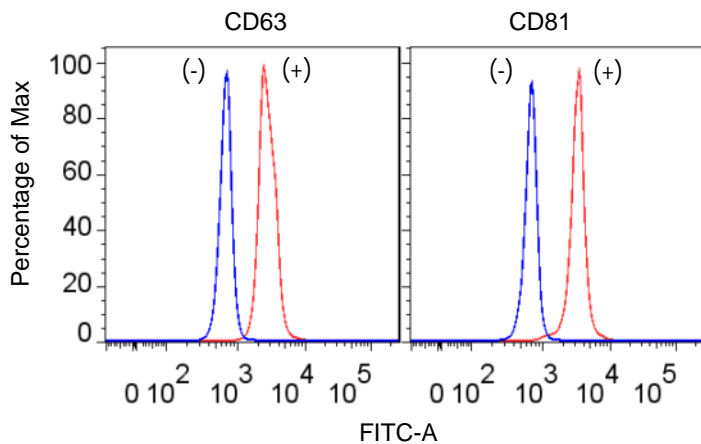

Supplement: Supplementary file 1 [file diagnostics-13-02968-s001.zip › Figure S2.pdf]

DAPI

Merge

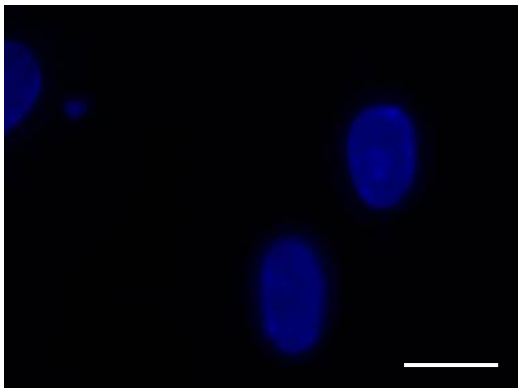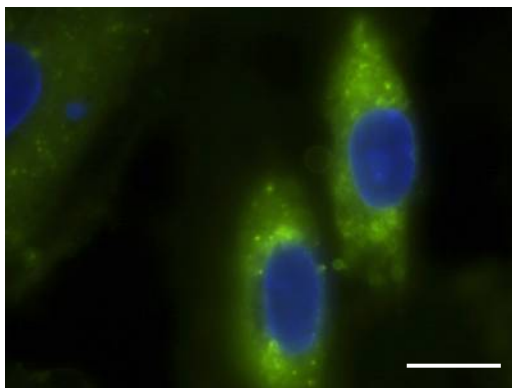

OSA exosomes

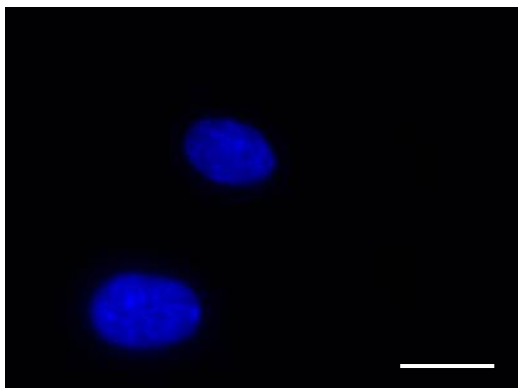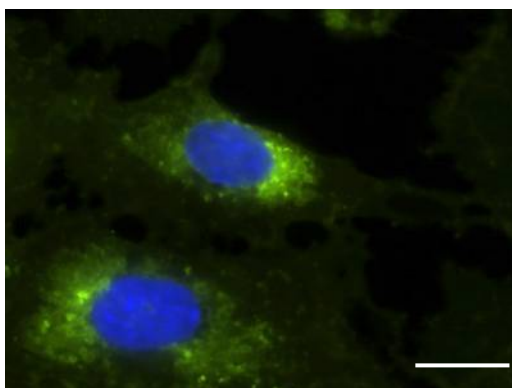

OSAT exosomes

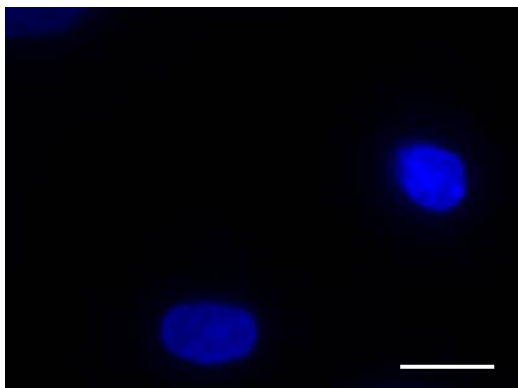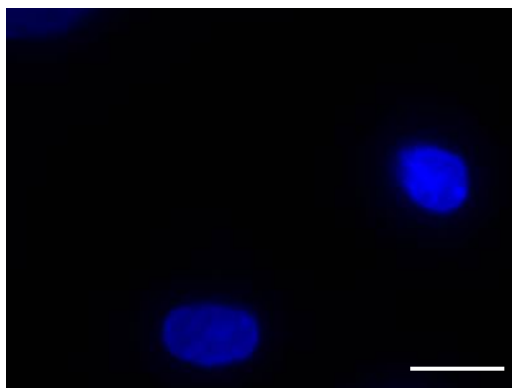

No exosomes

Supplement: Supplementary file 1 [file diagnostics-13-02968-s001.zip › Figure S3.pdf]

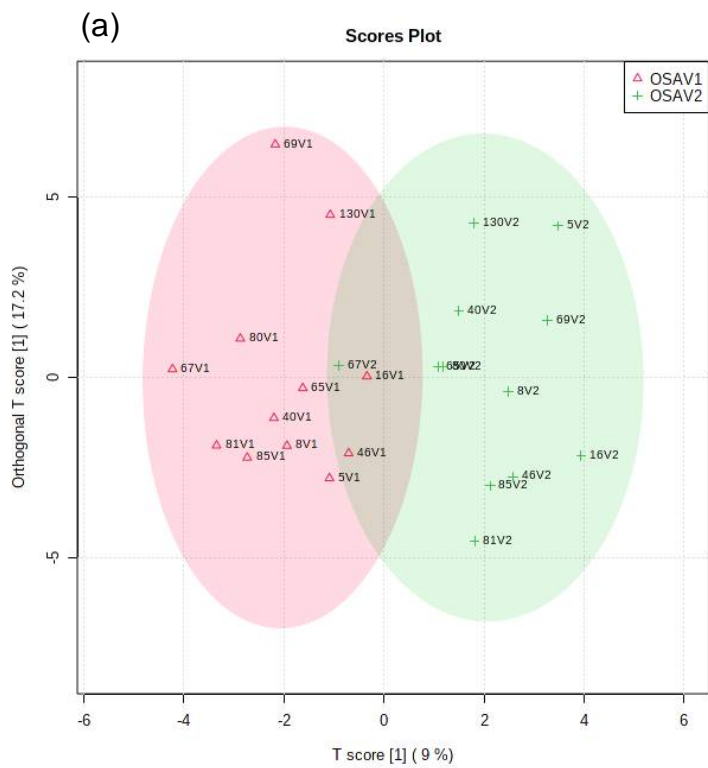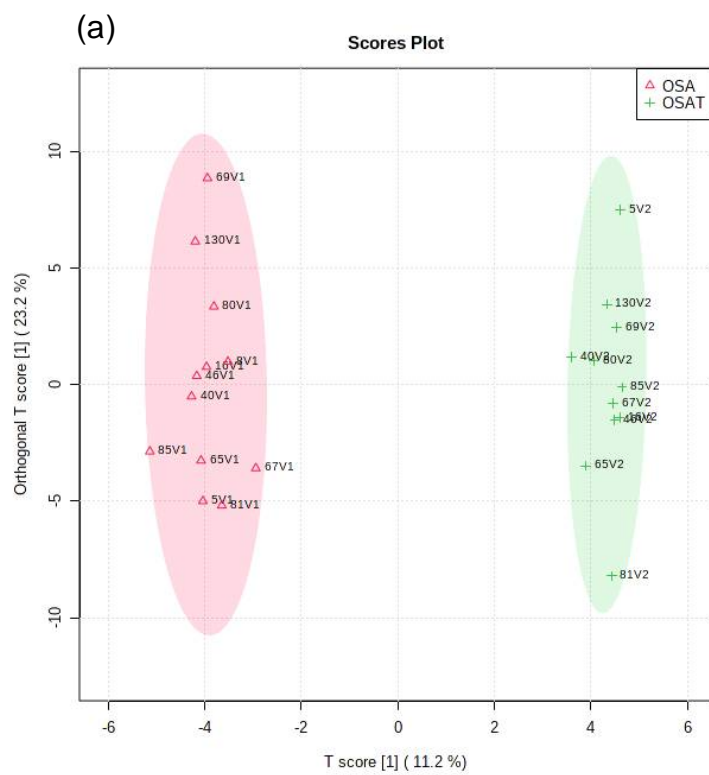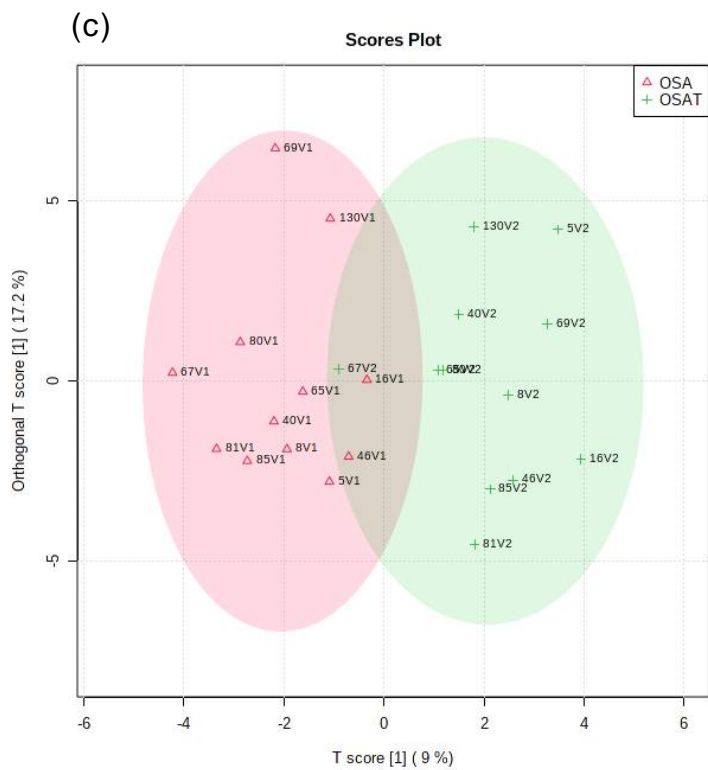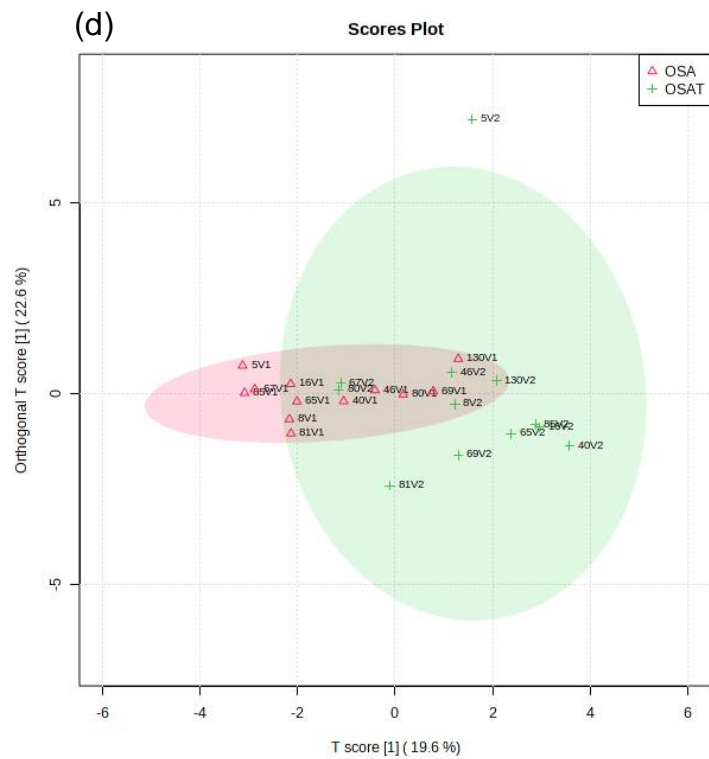

Supplement: Supplementary file 1 [file diagnostics-13-02968-s001.zip › Figure S4.pdf]

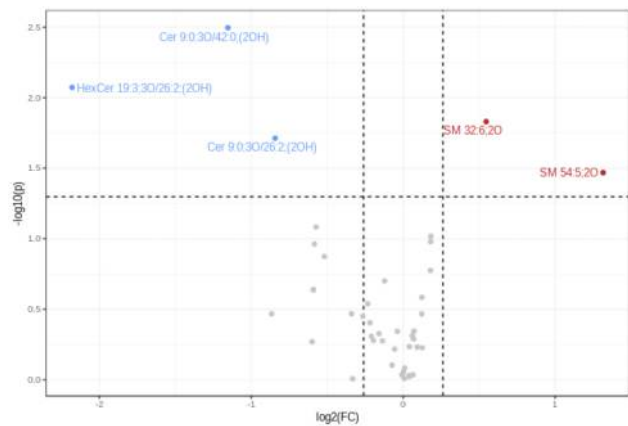

Status  
 DOWN  
 Non-SIG  
 UP

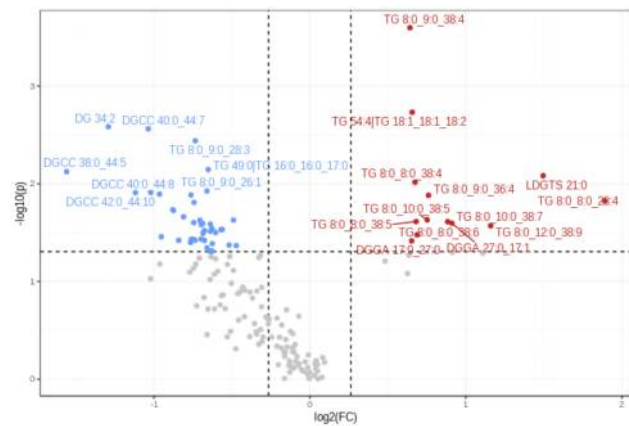

Status  
 DOWN  
 Non-SIG  
 UP

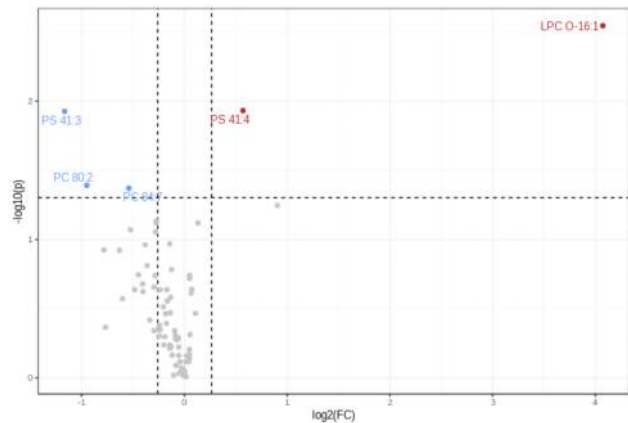

Status  
 DOWN  
 Non-SIG  
 UP

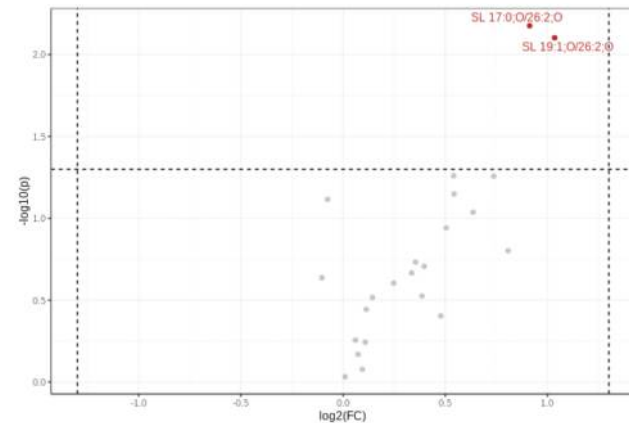

Status  
 Non-SIG  
 UP

Supplement: Supplementary file 1 [file diagnostics-13-02968-s001.zip › Figure S6.pdf]

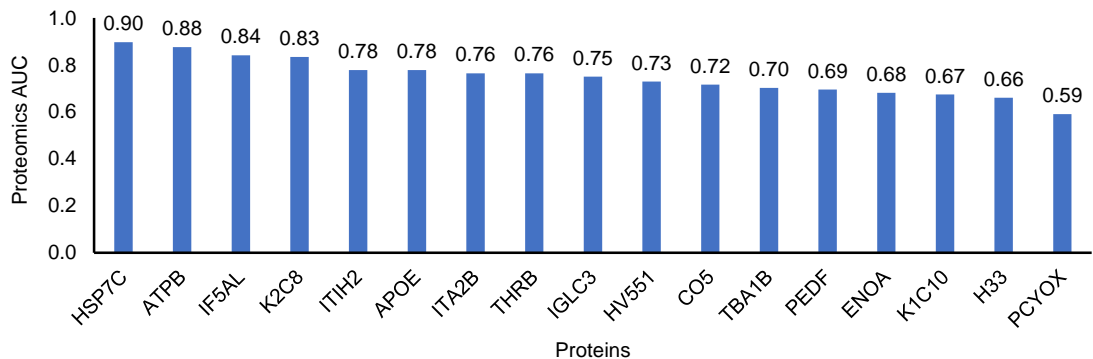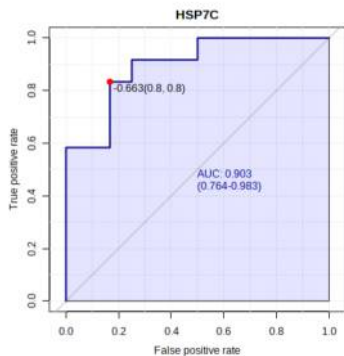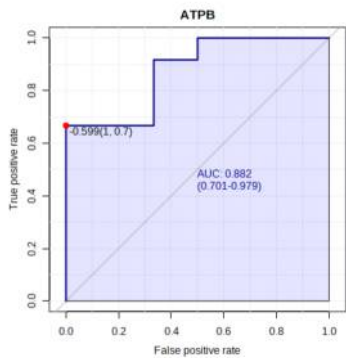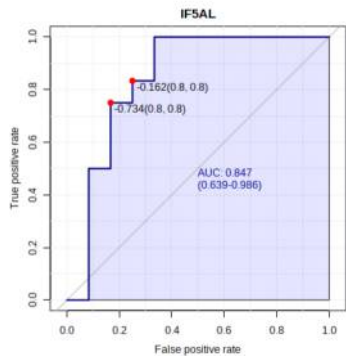

Supplement: Supplementary file 1 [file diagnostics-13-02968-s001.zip › Figure S7.pdf]

P-value

High

Low

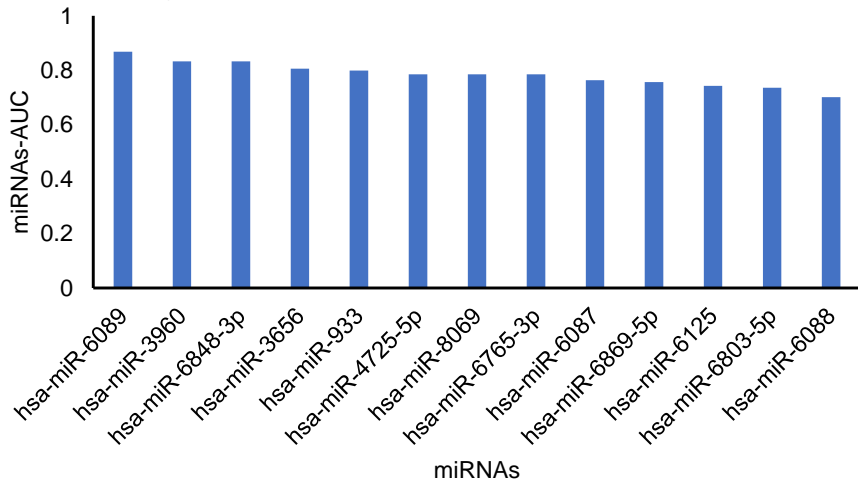

Supplement: Supplementary file 1 [file diagnostics-13-02968-s001.zip › Figure S8.pdf]

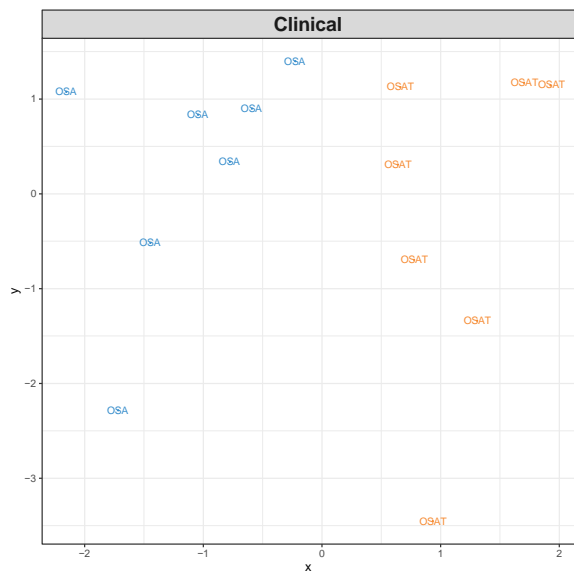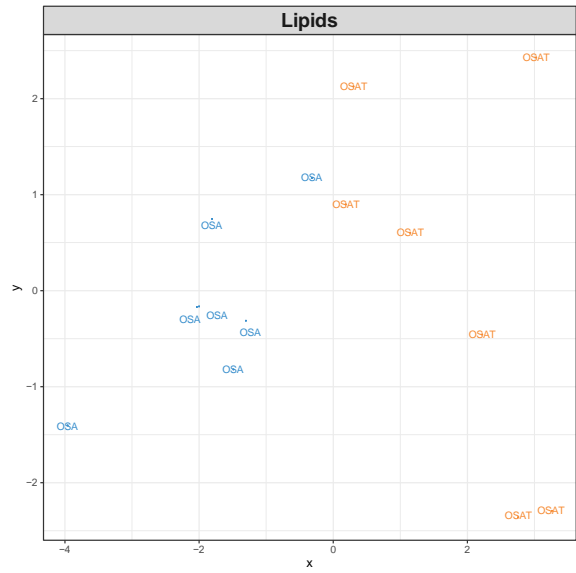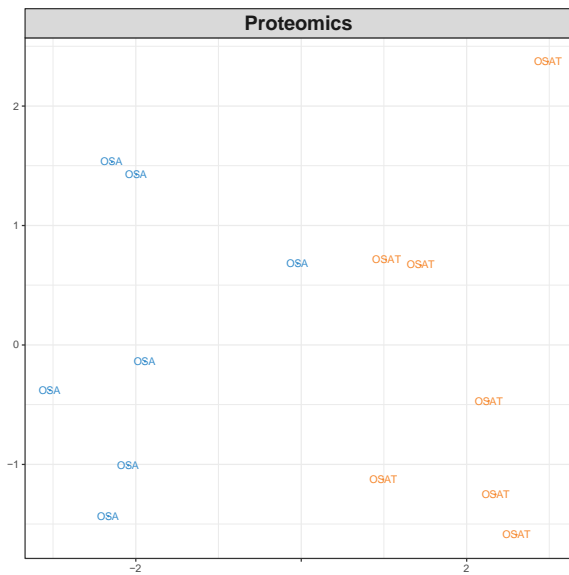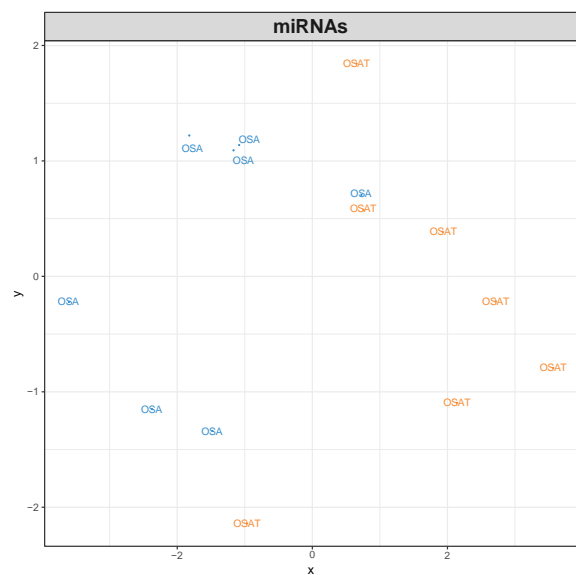

Supplement: Supplementary file 1 [file diagnostics-13-02968-s001.zip › Figure S9.pdf]
