# Supplementary material for: Assessing Bone Mineral Density in Weight-Bearing Regions of the Body through Texture Analysis of Abdomen and Pelvis CT Hounsfield Unit
Source: Diagnostics (Basel). 2023 Sep 16;13(18):2968. doi: 10.3390/diagnostics13182968 (PMC10529497; doi:10.3390/diagnostics13182968)
Supplement: Supplementary file 1 [file Figure S5.pdf › Figure S5.pdf]

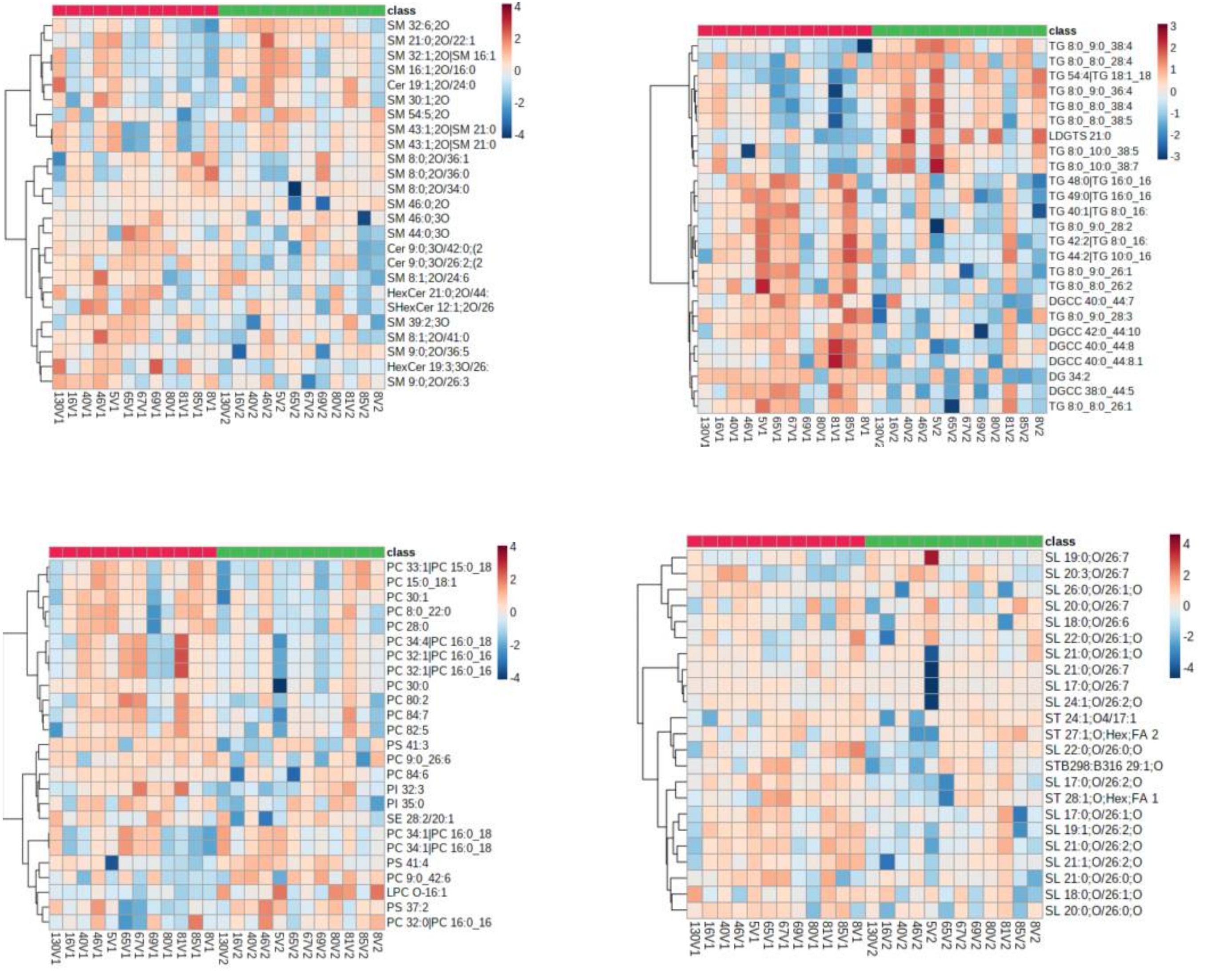
class
4
2
0
-2
-4
SM 32:6;2O
SM 21:0;2O/22:1
SM 32:1;2O|SM 16:1
SM 16:1;2O/16:0
Cer 19:1;2O/24:0
SM 30:1;2O
SM 54:5;2O
SM 43:1;2O|SM 21:0
SM 43:1;2O|SM 21:0
SM 8:0;2O/36:1
SM 8:0;2O/36:0
SM 8:0;2O/34:0
SM 46:0;2O
SM 46:0;3O
SM 44:0;3O
Cer 9:0;3O/42:0;(2
Cer 9:0;3O/26:2;(2
SM 8:1;2O/24:6
HexCer 21:0;2O/44:
SHexCer 12:1;2O/26
SM 39:2;3O
SM 8:1;2O/41:0
SM 9:0;2O/36:5
HexCer 19:3;3O/26:
SM 9:0;2O/26:3
130V1
16V1
40V1
46V1
5V1
65V1
67V1
69V1
80V1
81V1
85V1
8V1
130V2
16V2
40V2
46V2
5V2
65V2
67V2
69V2
80V2
81V2
85V2
8V2
class
3
2
1
0
-1
-2
-3
TG 8:0_9:0_38:4
TG 8:0_8:0_28:4
TG 54:4|TG 18:1_18
TG 8:0_9:0_36:4
TG 8:0_8:0_38:4
TG 8:0_8:0_38:5
LDGTS 21:0
TG 8:0_10:0_38:5
TG 8:0_10:0_38:7
TG 48:0|TG 16:0_16
TG 49:0|TG 16:0_16
TG 40:1|TG 8:0_16:
TG 8:0_9:0_28:2
TG 42:2|TG 8:0_16:
TG 44:2|TG 10:0_16
TG 8:0_9:0_26:1
TG 8:0_8:0_26:2
DGCC 40:0_44:7
TG 8:0_9:0_28:3
DGCC 42:0_44:10
DGCC 40:0_44:8
DGCC 40:0_44:8.1
DG 34:2
DGCC 38:0_44:5
TG 8:0_8:0_26:1
130V1
16V1
40V1
46V1
5V1
65V1
67V1
69V1
80V1
81V1
85V1
8V1
130V2
16V2
40V2
46V2
5V2
65V2
67V2
69V2
80V2
81V2
85V2
8V2
class
4
2
0
-2
-4
PC 33:1|PC 15:0_18
PC 15:0_18:1
PC 30:1
PC 8:0_22:0
PC 28:0
PC 34:4|PC 16:0_18
PC 32:1|PC 16:0_16
PC 32:1|PC 16:0_16
PC 30:0
PC 80:2
PC 84:7
PC 82:5
PS 41:3
PC 9:0_26:6
PC 84:6
PI 32:3
PI 35:0
SE 28:2/20:1
PC 34:1|PC 16:0_18
PC 34:1|PC 16:0_18
PS 41:4
PC 9:0_42:6
LPC O-16:1
PS 37:2
PC 32:0|PC 16:0_16
130V1
16V1
40V1
46V1
5V1
65V1
67V1
69V1
80V1
81V1
85V1
8V1
130V2
16V2
40V2
46V2
5V2
65V2
67V2
69V2
80V2
81V2
85V2
8V2
class
4
2
0
-2
-4
SL 19:0;O/26:7
SL 20:3;O/26:7
SL 26:0;O/26:1;O
SL 20:0;O/26:7
SL 18:0;O/26:6
SL 22:0;O/26:1;O
SL 21:0;O/26:1;O
SL 21:0;O/26:7
SL 17:0;O/26:7
SL 24:1;O/26:2;O
ST 24:1;O4/17:1
ST 27:1;O;Hex;FA 2
SL 22:0;O/26:0;O
STB298:B316 29:1;O
SL 17:0;O/26:2;O
ST 28:1;O;Hex;FA 1
SL 17:0;O/26:1;O
SL 19:1;O/26:2;O
SL 21:0;O/26:2;O
SL 21:1;O/26:2;O
SL 21:0;O/26:0;O
SL 18:0;O/26:1;O
SL 20:0;O/26:0;O
130V1
16V1
40V1
46V1
5V1
65V1
67V1
69V1
80V1
81V1
85V1
8V1
130V2
16V2
40V2
46V2
5V2
65V2
67V2
69V2
80V2
81V2
85V2
8V2
